# Assessment of threat and negativity bias in virtual reality

**DOI:** 10.1038/s41598-020-74421-1

**Published:** 2020-10-15

**Authors:** Christopher Baker, Ralph Pawling, Stephen Fairclough

**Affiliations:** School of Psychology, Liverpool John Moores University, Liverpool, UK

**Keywords:** Psychology, Human behaviour, Psychiatric disorders, Emotion, Information technology, Physiology

## Abstract

Negativity bias, i.e., tendency to respond strongly to negative stimuli, can be captured via behavioural and psychophysiological responses to potential threat. A virtual environment (VE) was created at room-scale wherein participants traversed a grid of ice blocks placed 200 m above the ground. Threat was manipulated by increasing the probability of encountering ice blocks that disintegrated and led to a virtual fall. Participants interacted with the ice blocks via sensors placed on their feet. Thirty-four people were recruited for the study, who were divided into High (HN) and Low (LN) Neuroticism groups. Movement data were recorded alongside skin conductance level and facial electromyography from the corrugator supercilii and zygomaticus major. Risk-averse behaviours, such as standing on ‘safe’ blocks and testing blocks prior to movement, increased when threat was highest. HN individuals exhibited more risk-averse behaviour than the LN group, especially in the presence of high threat. In addition, activation of the corrugator muscle was higher for HN individuals in the period following a movement to an ice block. These findings are discussed with respect to the use of room-scale VE as a protocol for emotion induction and measuring trait differences in negativity bias within VR.

## Introduction

A number of experimental methods have been developed to induce specific emotional states in human participants^1^ including specialized media databases^2,3^ and the use of confederates^4^. Virtual Reality (VR) is a recent addition to this repertoire^5^, capable of inducing a range of different emotions, from feelings of fear due to phobia^6^ and social anxiety^7^ to the restorative experience of sitting in a virtual forest^8^. The potency of these experiences depends on the technical capacity of the VR system to create a sense of presence^9,10^.

Previous VR-based experiments have induced emotional states by manipulating the ambient atmosphere of the Virtual Environment (VE)^11–14^ using lighting and weather effects. Others stimulated negative emotions by creating an illusion of height in VR^15–22^; the effectiveness of virtual height to induce fear and anxiety is enhanced by natural sensorimotor contingencies^23^ (i.e. control of avatar using feet/hands) and the incorporation of physical props to mimic virtual objects, e.g., wooden plank^16,20,21^. A number of these studies also incorporated measures of psychophysiology^16,18–20,22^ and neurophysiology^21^ into their protocols.

The illusion of height is an example of a threat stimulus^24^ that elicits anxiety in the majority of people. Negative emotional states, such as anxiety, incorporate predispositions for specific types of actions^25,26^; for example, increased levels of anxiety predispose the organism towards behaviours associated with avoidance^27^, e.g., move away from the edge of a high roof, walk slowly when there is a risk of falling. These behavioural responses are not ‘simple’ reflex actions but can be nuanced, flexible and sensitive to context^28^. For example, the Evaluative Space Model^29,30^ (ESM) describes a conceptual space wherein dynamic and flexible responses to emotional stimuli are driven by the relative activation of two superordinate dimensions of positivity and negativity. According to this model, the strength of activation on positivity and negativity dimensions relates to the extremity of the stimulus and incorporates a negativity bias^31^ wherein negative inputs exert a stronger effect on emotional/behavioural outputs than their positive counterparts. Therefore, if the level of threat in an environment is increased in a linear fashion, we should observe a disproportionate increase of anxiety and avoidant behaviour when threat is perceived to be moderate or high. For example, Biedermann et al. reported slower movement and less time spent in those areas of an elevated virtual maze that were associated with higher subjective anxiety^20^.

Similarly, increased activation of the negativity dimension is associated with changes in emotional expression at higher levels of threat, which can be measured implicitly using facial ElectroMyoGraphy (fEMG)^32,33^. Activation of the corrugator supercilia and zygomaticus major reliability index positive and negative valence respectively^34–36^ and are correlated with self-reported affective states^33,37^. In this case, increased activation of the negativity dimension enhances corrugator reactivity and suppresses activation of the zygomaticus. In addition, increased negativity would precipitate activation of the sympathetic branch of the autonomic nervous system, e.g., increased skin conductance level (SCL) is associated with arousal during emotional experiences^38–40^.

To summarise, a higher level of threat leads to inflated changes in avoidant behaviour and psychophysiological markers of negative affect and autonomic activation. There is also evidence for stable trait differences in this negativity bias across a range of experimental paradigms^41,42^. Heightened sensitivity to negative stimuli is a characteristic associated with increased trait neuroticism^43^ and high neuroticism has also been associated with a ‘harm avoidant’ style of coping^44^, leading to exaggerated psychophysiological reactivity to increased threat^45^ and negative images and films^46,47^. Hence, the accelerated gradient of negativity and its disproportionate impact on behaviour and psychophysiology may be augmented for those with high trait neuroticism who are predisposed towards negative affectivity.

The goal of the current study was to explore behavioural and psychophysiological responses to systematic increases of threat within a room-scale VE. Participants were required to negotiate a virtual variant on the visual cliff^48^ where they must traverse a grid of large, translucent blocks of ice that floated in the air at a minimum height of 200 m. Participants negotiated three levels of the VE from low to high threat. Patterns of movement were recorded alongside ambulatory psychophysiology, e.g., fEMG, SCL. It was predicted that movement patterns would reflect greater caution and risk aversion as threat increased. It was also hypothesised that increased threat would provoke higher sympathetic activation in the SCL and greater negative valence (increased activation of corrugator and reduced activation of zygomaticus). It was predicted that participants with higher trait neuroticism would exhibit a greater tendency towards risk-averse behaviour and higher psychophysiological reactivity to threat (e.g., higher activation/negative valence).

## Methods

### Participants

Thirty-four participants (21 female) were recruited with a mean age of 23.72 years (SD = 3.15). Participants were recruited from a university population of postgraduates and undergraduates. The only criterion for inclusion was that participants must be aged over 18 years and able to walk without assistance. Participants were excluded if they were currently taking any medication. Participants completed the OCEAN.20 personality inventory^49^ and the Situational Vertigo Questionnaire (SVQ) questionnaire^50^. The OCEAN.20 inventory includes twenty items, five of which are each dedicated to each of the Big Five personality traits^51^, e.g., Openness, Conscientiousness, Extraversion, Agreeableness, Neuroticism. For the purpose of the current study, we only measured trait Neuroticism where participants provided responses on a 7-point Likert scale (1 = very strongly disagree; 0 = neutral; 7 = very strongly agree) to five statements, e.g., ‘My feelings are easily hurt’ ‘I am often nervous and tense’. The reliability of this trait as measured on the OCEAN.20 and indexed by Cronbach’s alpha was 0.81. The SVQ includes 19 items designed to capture visual vertigo with a Cronbach’s alpha score of 0.96^52^; participants are asked to rate the prevalence of vertigo symptoms on a 4-point scale from 0 (not at all) to 4 (very much) in a number of scenarios, e.g. ‘riding as a passenger in a car on winding or bumpy roads’ ‘standing in a lift as it stops’.

Participants were divided into High Neuroticism (HN) and Low Neuroticism (LN) groups using a median split of 24; each group consisted of 16 participants (2 participants fell on the median and were omitted from further analyses). The HN group included 10 females with a mean age of 23.76 years (SD = 3.53), whereas the LN group contained 11 females with a mean age of 24.69 years (SD = 2.83). Between-group t-tests confirmed: (i) no significant differences in age, (ii) no significant differences in scores on the SVQ, i.e., HN group (M = 1.38, SD = 0.26), LN group (M = 1.41, SD = 0.46), and (iii) trait neuroticism was significantly higher for the HN group (M = 29.94, SD = 3.87) compared to the LN group (M = 17.94, SD = 3.71), [t(30) = 8.95, *p* < 0.001]. The experimental protocol was approved by the Liverpool John Moores University Research Ethics Committee prior to data collection (Ref: 19/NSP/006), which operates within guidelines from the UK Research Integrity Office Code of Practice for Research and in accordance with the Declaration of Helsinki.

### Virtual environment

**Figure 1:**
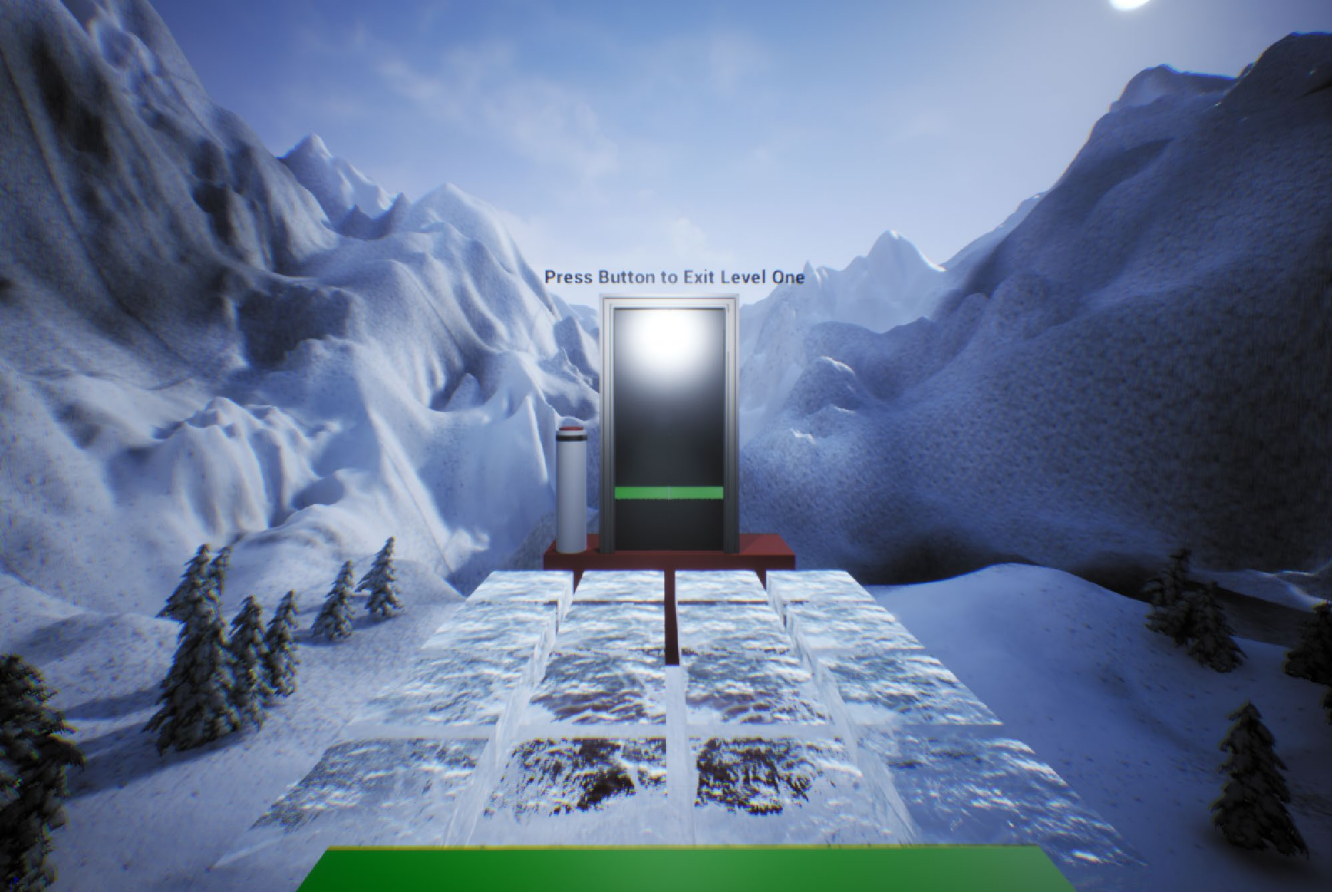
View of the virtual environment from perspective of participant.

**Figure 2:**
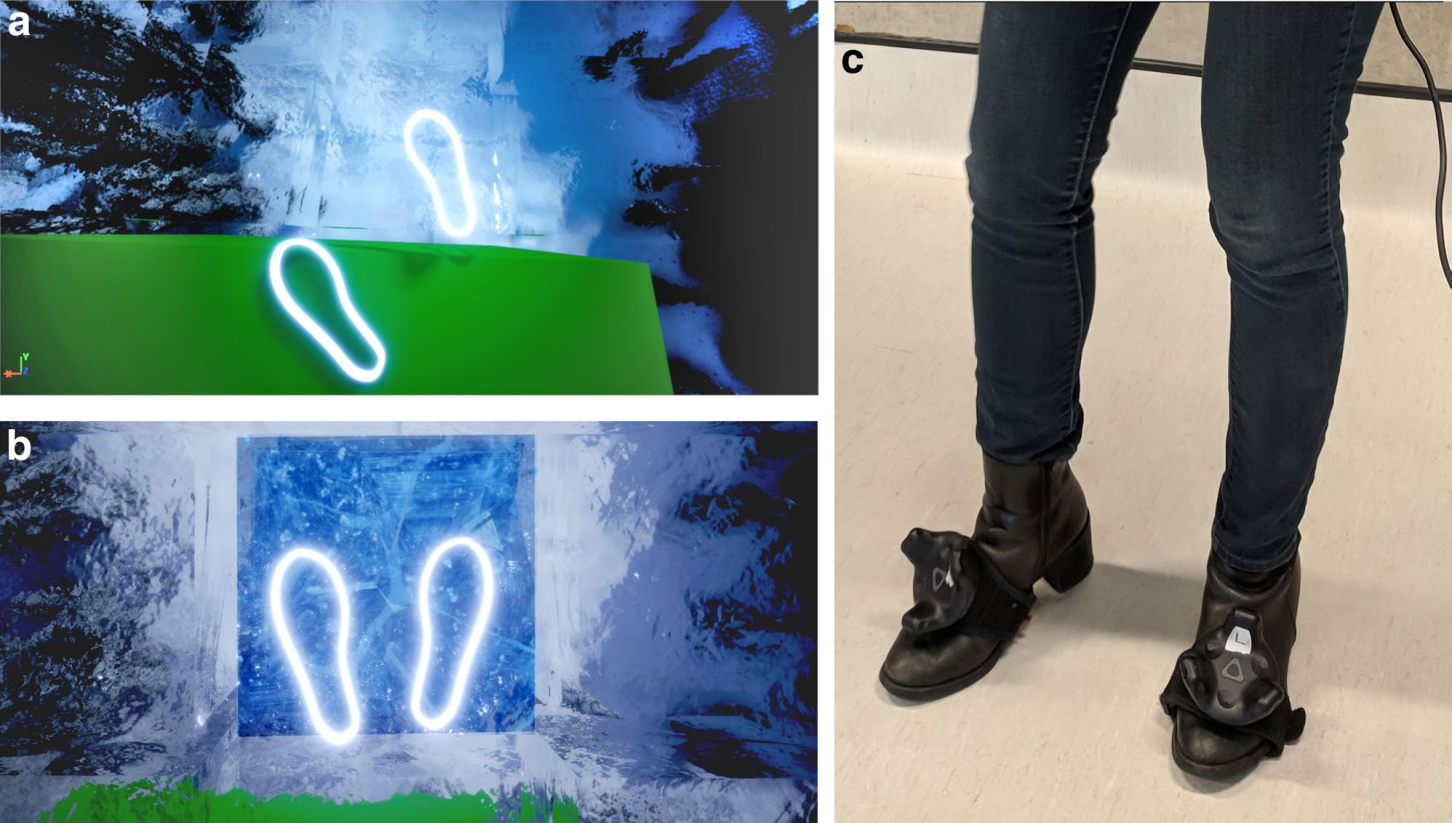
Anti -clockwise from left, (**a**) One-footed movement to Solid block (**b**) Two-feet movement to Crack block, and (**c**) Participant wearing VIVE Trackers on feet.

Participants were required to traverse a grid of 4 × 4 ice blocks from a start platform to a goal platform where an automatic door could be activated (Fig. 1). Each block was approximately 70 × 70 cm. The ice blocks were suspended in the air at a virtual height of 200 m and participants interacted with them via foot movements, achieved by attaching sensors to participants’ feet in addition to conventional handheld trackers (Fig. 2c). Foot sensors allowed participants to interact with ice blocks in two ways: (1) a one-footed testing movement (Risk Assessment) (Fig. 2a) or (2) a two-feet movement in which participants committed to standing on the block (Risk Decision) (Fig. 2b).

The grids of ice blocks contained three types of block. If the block was Solid, it would support the weight of the participant and did not change appearance when activated. Crack blocks also supported the weight of the participant but any interaction caused a change of colour from translucent to blue accompanied by a cracking sound effect 500 ms after activation (Fig. 2b). Fall blocks behaved in exactly the same fashion as Crack blocks, but any two-feet activation triggered a shattering sound effect after 500 ms when the block would disintegrate and participants experienced a virtual fall (a video of the VE is provided in the supplementary materials). In the event of a fall, participants were required to return to the start position and repeat their journey across the grid; a gap would appear in the grid to indicate the former position of the Fall block.

**Figure 3:**
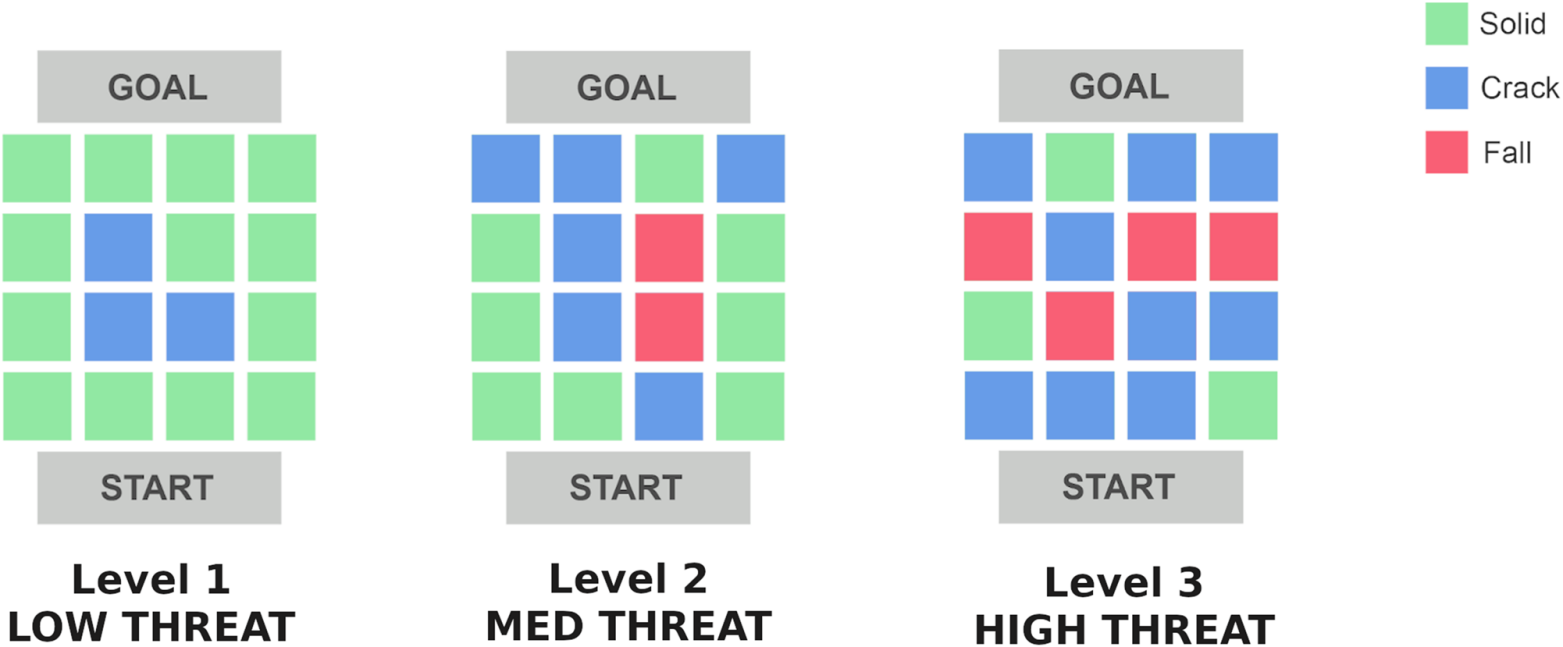
Layout of the ice block grid for all three levels of threat used in the experiment. See text in Virtual Environment for further explanation.

Participants were required to traverse three different grids or Levels during the experiment. The level of threat was manipulated by increasing the number of Crack and Fall blocks from Level 1 to Level 3 (Fig. 3). In order to complete each Level, participants activated an automatic door (Fig. 1) with one of the handheld controllers. At the end of level 1, participants would be instructed to turn and return to the start location for level 2. This process was repeated at the end of level 2.

### Virtual reality system

The experiment was conducted within a physical space of 5 × 4 m. Participants wore a tethered HTC Vive head-mounted display (HMD) with two base stations positioned in diagonally-opposite-corners of the space. Each participant held two hand controllers and two trackers were attached to their feet. Hand and feet positions were represented as white luminous outlines in the VE (Fig. 2a, b). The VE was constructed in Unreal Engine 4.21. All assets were purpose built for the study. The VE was rendered on a desktop PC with custom C++ code integrated directly into the Unreal Engine system to capture interactions with blocks and recorded timings. Data representing position of the head, hands and feet were recorded at a rate of 5 Hz and logged to a text file.

### Movement

The primary mode of interaction within the VE was achieved via foot movements. By monitoring the position of each foot and interaction with custom trigger volumes in the VE, the frequency of one-footed and two-footed movements were recorded and cross-registered with a position within the VE. Both frequency and timings of all interactions were recorded, e.g., number of one-footed or two-feet interactions with Solid, Crack and Fall blocks. Therefore, we could calculate the frequency of Risk Assessment (one-footed movement) and Risk Decision (two-feet movement) interactions with each block type. Timing data were also logged, e.g., amount of time spent standing on each block.

### Event-related psychophysiology

Facial electromyography (fEMG) was recorded at 1000 Hz from the corrugator supercilii and zygomaticus major via the Bionomadix ambulatory psychophysiology system (BIOPAC). fEMG data were processed as follows: (1) bandpass filter applied at 49-51 Hz to remove 50 Hz noise, (2) filtered between 20 and 400 Hz, (3) rectified and smoothed via linear envelope (9 Hz filter), and (4) subjected to root mean square transformation^49,50^ . Skin Conductance Level (SCL) was also recorded at 1000 Hz via the Bionomadix system. Data were collected from the index finger and second digit of the non-dominant hand and subsequently filtered with a high pass filter at 0.05 Hz.

The level of activation from all psychophysiological data were calculated on an event-related basis. For each one-footed and two-feet interaction with a Solid or Crack block, psychophysiological data were averaged during a pre-movement period of 750 ms and a post-movement period of 1500 ms duration. These data were averaged across all events and baselined (i.e., pre-movement values were subtracted from post-movement values) create Grand Averages for the 0–750 ms and 751–1500 ms periods following each movement for: (1) each category of interaction (Risk Assessment/Risk Decision), and (2) two types of Block (Solid vs. Crack). In order to maximise the number of interactions that contributed to each Grand Average, the level of the VE was omitted from the analyses of psychophysiological data.

### Procedure

Participants arrived at the laboratory, they read a Participant Information Sheet and provided informed consent. Participants completed the questionnaires and received written instructions about the task and the VE. The fEMG sensors were attached to the corrugator and zygomaticus sites and SCL sensors taped to the second phalanx of fingers on the non-dominant hand. Participants were subsequently fitted with the Vive Tracker sensors on their feet, the HMD and the hand controllers. The VE for Level 1 was activated and participants were provided with a 30 s countdown while standing on the platform before the grid appeared. Once the grid was available, participants could progress at their own speed. When they reached the task goal (the automatic door), participants received a transition cue to return to the starting position for Level 2 and the process was repeated. At the end of Level 2, the same procedure was followed for Level 3. Upon completion of Level 3, the VR apparatus and psychophysiological sensors were removed and participants were thanked for their time and debriefed.

### Hypotheses and statistical analyses

It was hypothesised that movement would be increasingly risk-averse as threat was increased from Level 1 to Level 3, e.g., greater frequency of Risk Assessments, longer time spent standing on Solid blocks. It was predicted that this pattern of risk-averse movement would be more pronounced for HN compared to LN individuals. These effects were assessed via 2 (HN vs. LN) × 3 (Level) × 2 (Risk Assessment vs. Decision) × 2 (Solid vs. Crack Block) ANOVA model.

Psychophysiological data were collected on an event-related basis for each type of interaction. It was hypothesised that highest levels of corrugator and SCL activation plus lower levels of zygomaticus activity would be observed after two-footed interactions with Crack blocks, which contained the greatest potential for a fall. It was also predicted that this pattern would be enhanced for HN individuals compared to those in the LN group. Each psychophysiological grand average was analysed via a 2 (HN vs. LN) × 2 (Risk Assessment vs. Decision) × 2 (Solid vs. Crack Block) × 2 (post-movement periods: 0–750 ms vs. 751–1500 ms) ANOVA.

All statistical analyses were conducted using SPSS v.26. Outliers were defined as any value that deviated from the mean for that cell by 3 or more standard deviations. In the case of within-participants comparisons, sphericity was assessed via Mauchly’s Test and the Greenhouse–Geisser adjustment was applied.

## Results

### Movement

The analyses of participants’ movements were based exclusively on interactions with Solid and Crack blocks. Fall blocks were included simply to increase perceptions of threat during Level 2 (L2) and Level 3 (L3) in the VE (Fig. 1). Twenty out of our 34 participants triggered at least one Fall block at either L2 or L3.

### Mean duration

An analysis of mean duration revealed a significant main effect for Level [F(2,29) = 7.95, p = 0.002, eta^2^ = 0.35], i.e., participants spent longer standing on blocks at L3 (M = 13.93 s, SD = 9.62) compared to L1 (M = 7.90 s, SD = 4.54) or L2 (M = 8.24 s, SD = 5.00). In addition, participants spent longer standing on Solid blocks (M = 11.13 s, SD = 6.21) compared to Crack blocks (M = 8.94 s, SD = 5.14) [F(1,30) = 5.53, p = 0.025, eta^2^ = 0.16]. There was also a significant main effect for neuroticism [F(1,30) = 4.89, p = 0.035, eta^2^ = 0.14]; individuals in the HN group stood on blocks for longer (M = 12.00 s, SD = 7.42) compared to the LN group (M = 8.04 s, SE = 7.33).

**Figure 4:**
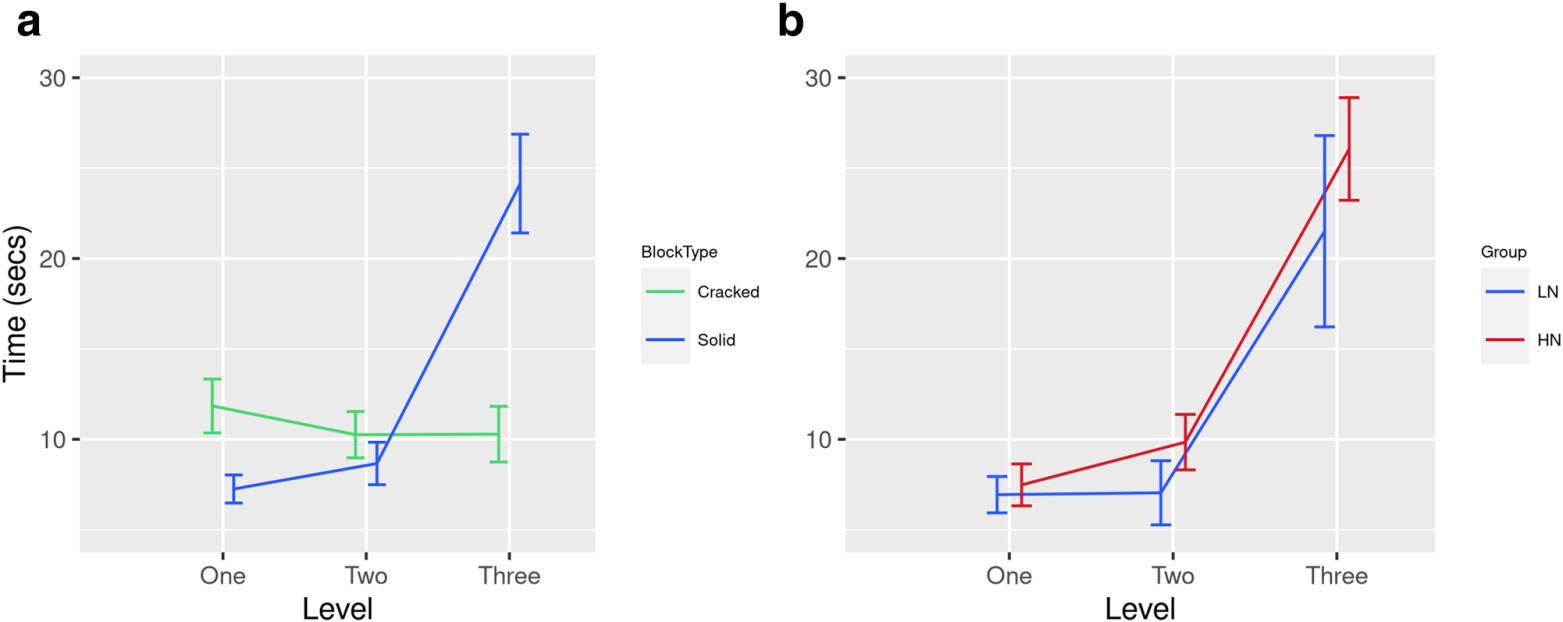
Timings data for two interaction effects: (**a**) Level × block type, and ( × ) Neuroticism × Level at L3 for Solid Blocks only.

A significant interaction between Level x Block [F(2,29) = 13.06, p < 0.001, eta^2^ = 0.47] indicated that participants spent significantly longer on Solid blocks (M = 19.14 s, SE = 2.73) compared to Crack blocks (M = 8.82 s, SE = 1.21) during L3 [t(31) = 4.34, p = 0.011] (Fig. 4a). There was also a significant interaction between Neuroticism x Block [F(1,30) = 6.01, p = 0.020, eta^2^ = 0.17], i.e., HN group spent longer on the Solid blocks (M = 14.14 s, SD = 9.03) compared to LN individuals (M = 7.94 s, SD = 9.01). A significant 3-way interaction [F(2,29) = 3.52 , p < 0.043, eta^2^ = 0.20] revealed that the HN group spent longer standing on Solid blocks (M = 27.44 s, SD = 20.91) compared to LN individuals (M = 10.83 s, SD = 20.93) but only during L3 (Fig. 4b). In summary, when the threat level of the VE was highest, participants spent longer standing on Solid compared to Crack blocks and this effect was particularly pronounced for HN individuals.

### Frequency of interactions

**Figure 5:**
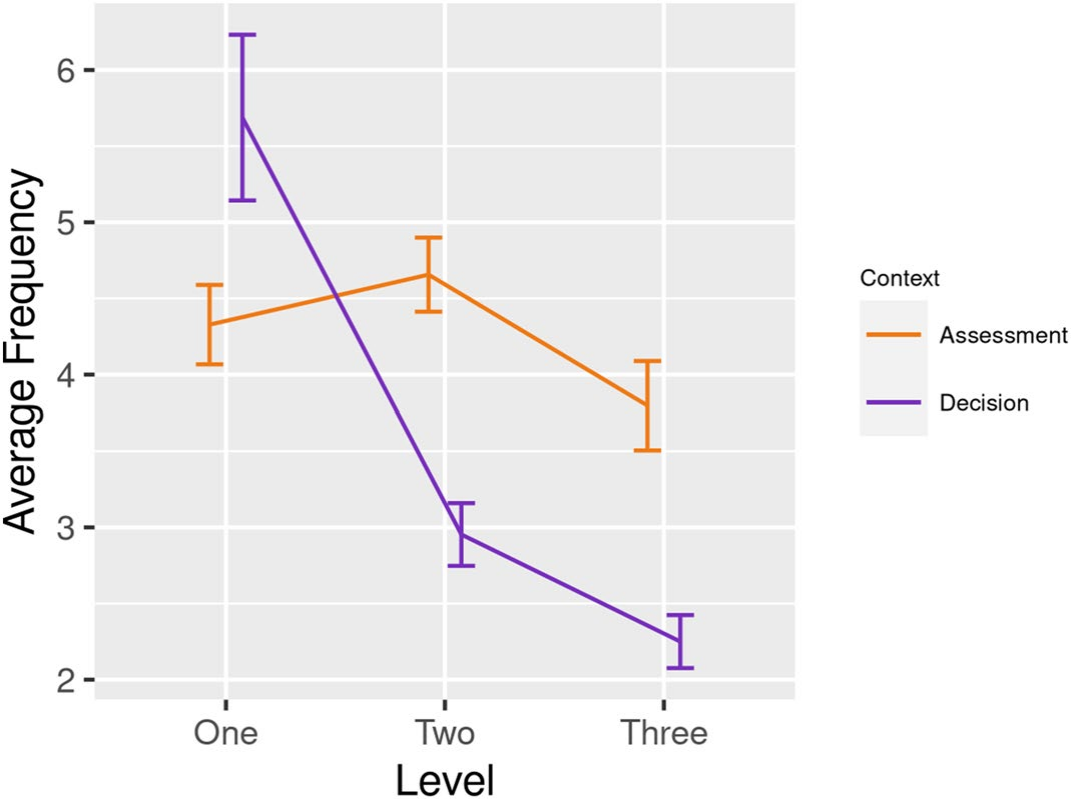
Average frequency of Risk Assessments (one-foot interactions) and Risk Decisions (two-feet interactions) across three levels of the experiment.

The frequency data captured the number of times each participant interacted with blocks in the VE. The Context of each interaction was categorised as Risk Assessment (testing the block with one foot) or Risk Decision (stepping onto the block with two feet). The ANOVA model revealed a significant main effect for Context [F(1, 29) = 11.31 , p = 0.006, eta^2^ = 0.28], i.e., the frequency of Risk Assessments (M = 4.19, SE = 0.18) was greater than Risk Decisions (M = 3.61, SE = 0.18). The significant main effect for Block [F(1, 29) = 31.28, p = 0.002, eta^2^ = 0.52] demonstrated that participants interacted with Solid blocks (M = 4.46, SD = 1.11) more frequently than Crack blocks (M = 3.33, SD = 1.12). There was a significant interaction between Context x Level [F(2, 58) = 49.78 , p = 0.001, eta^2^ = 0.63]; the frequency of Risk Decisions was greater than Risk Assessments during Level 1 [t(31) = -2.14, p = 0.044] but this trend reversed during L2 [t(32) = 5.25, p = 0.009] and L3 [t(31) = 4.24, p = 0.008] (Fig. 5).

**Figure 6:**
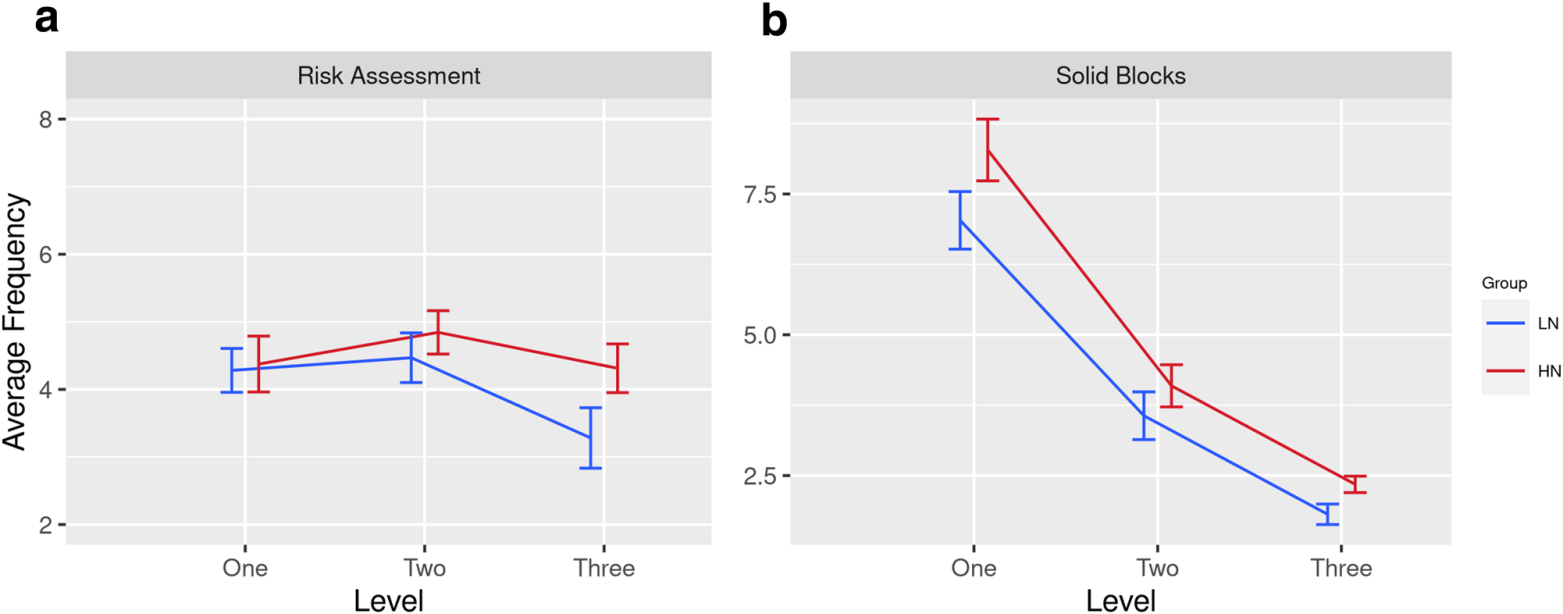
**(a)** average frequency of Risk Assessments for HN and LN participants across all Levels of VE, (**b**) average frequency of interactions with Solid blocks for HN and LN participants across all Levels.

The ANOVA also revealed a significant three-way interaction between Context x Level x Neuroticism [F(2,28) = 3.14, p = 0.052, eta^2^ = 0.10]. Post-hoc t-tests revealed a higher number of Risk Assessments for the HN group compared to the LN group, but only during level 3 [t(30) = 2.69, p = 0.014] as illustrated in Fig. 6a. A second significant 3-way interaction was observed between Block x Level x Neuroticism [F(2,28) = 9.61, p = 0.007, eta^2^ = 0.25]. Post-hoc tests indicated that HN individuals interacted more frequently with Solid blocks than LN individuals, but this difference only achieved significance at Level 1 [t(30) = 1.79, p = 0.052], see Fig. 6b. In summary, the frequency of Risk Assessments (testing blocks with one foot) increased with the threat level of VE and this movement was associated with higher trait neuroticism at L3. In addition, individuals in the HN group interacted more frequently with Solid blocks, particularly at L1.

### Psychophysiology

All psychophysiological data were subjected to 2 (High/Low Neuroticism) × 2 (Solid/Crack Block) × 2 (Pre/Post movement period).

### Skin conductance level (SCL)

SCL data were lost from three participants due to physical artifacts caused by contact between the sensor and the handheld controller. No statistically significant effects were found during analyses of SCL during Risk Assessment. The analyses of SCL activation during Risk Decision revealed only a significant main effect for Movement period [F(1,27) = 4.12, p = 0.053, eta^2^ = 0.13], i.e. SCL increased during the later post-movement period compared to the earlier period.

### Facial electromyography (fEMG)

**Figure 7:**
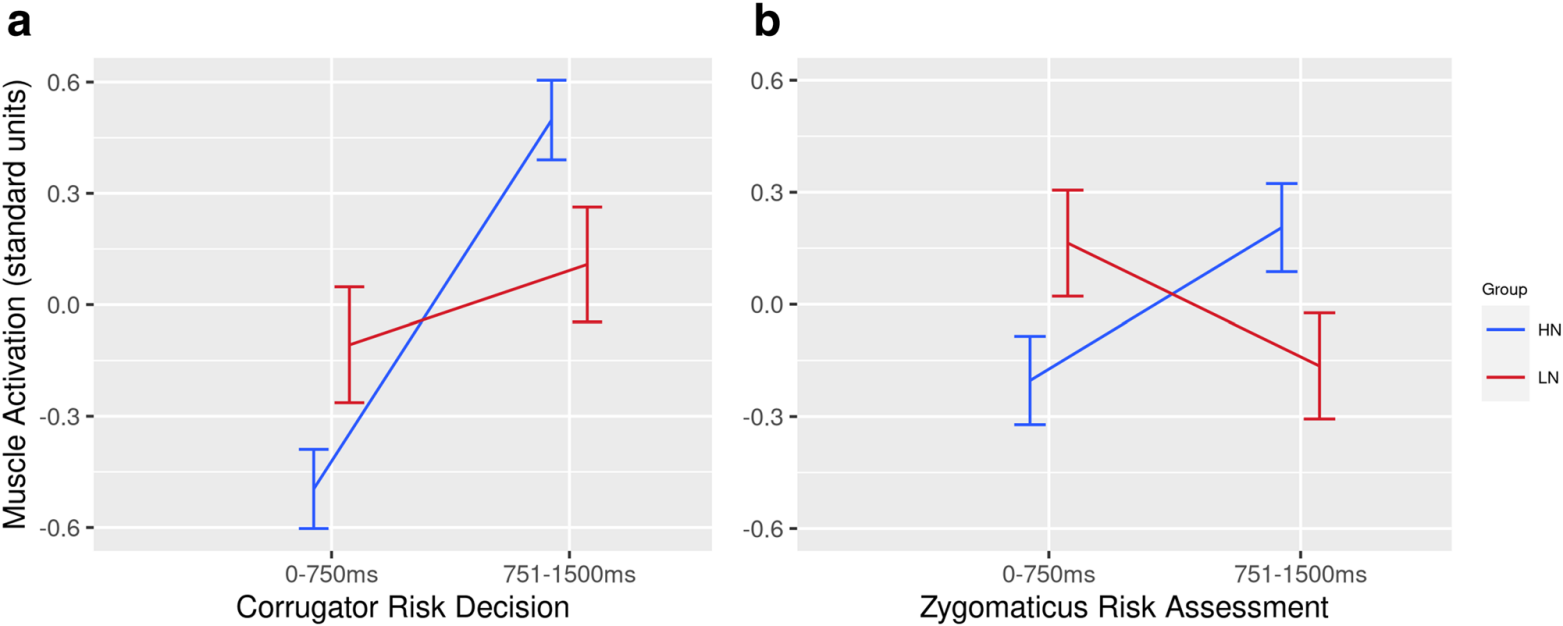
(**a**) mean activation of corrugator supercilii during second post-event period (751–1500 ms) for Risk Assessment interactions with Solid and Crack blocks, (**b**) mean activation of zygomaticus major between the first and second post-event periods when participants interacted with a Crack block.

An analysis of corrugator activation was conducted for Risk Assessment but no significant effects were found. The same ANOVA model was applied to corrugator data from Risk Decision, which revealed a marginal main effect for Block [F(1, 31) = 3.56, p = 0.062, eta^2^ = 0.10], a significant effect for Period [F(1, 31) = 10.51, p = 0.002, eta^2^ = 0.25], and a significant interaction between Neuroticism Group x Period [F(1, 31) = 4.34, p = 0.047, eta^2^ = 0.12]. The significant effect for Period indicated that corrugator activation increased in the 751-1500 ms period after the two-feet movement compared to the 0-750 ms period. The marginal Block effect suggested that stepping onto a Crack block with two-feet caused higher activation of the corrugator muscle compared to the same movement to a Solid block. The interaction between Group x Period is illustrated in Fig. 7a. Post-hoc t-tests revealed that corrugator activation was significantly higher during 751-1500 ms period following a two-feet interaction compared to the earlier period, but only for the HN group [t(16) = 3.39, p = 0.014].

The analysis of zygomaticus activation during Risk Assessment revealed only a significant interaction between Neuroticism Group × Period [F(1, 31) = 4.03, p = 0.053, eta^2^ = 0.12]. This interaction is illustrated in Fig. 7b. Post-hoc t-tests revealed significant between-group differences for both 0–750 ms [t(31) = 1.99, p = 0.051] and 751–1500 ms periods after movement occurred [t(31) = − 2.02, p = 0.052], i.e., greater zygomaticus activation during the 0–750 ms period for the LN group and greater activation for the HN group during the 751 = 1500 ms period (Fig. 7b). The same model was applied to zygomaticus data during Risk Decision, but no significant effects were found.

To summarise the fEMG analyses, activation of the corrugator muscle was associated with Risk Decision interactions, especially to Crack blocks when the risk of a virtual fall was highest. Individuals with high trait neuroticism tended to exhibit greater corrugator activation in the 715–1500 ms period after this interaction. When participants made a Risk Assessment, we found a pattern of individual differences wherein HN individuals showed greater activation of the zygomaticus during the period 751–1500 ms after one-footed movement compared to the LN group, this pattern was reversed during the 0–750 ms period.

## Discussion

Our analyses of behavioural data revealed evidence of risk aversion in response to increased threat and trait neuroticism, specifically:

- Participants spent longer standing on Solid vs. Crack blocks because the former were deemed to be ‘safe’ and this effect was pronounced at L3 (Fig. 4a).
- The number of Risk Assessments exceeded the frequency of Risk Decisions as participants progressed from L1 to L3 (Fig. 5). This pattern indicated greater caution as one-footed checks were used to identify Crack blocks.
- This pattern of risk-averse behaviour was enhanced for participants with higher trait neuroticism: (i) HN individuals spent longer standing on Solid blocks during L3 (Fig. 4b), (ii) participants in the HN group made more frequent Risk Assessments at L3 (Fig. 6a), and (iii) when Solid blocks were available at L1, HN individuals interacted more frequently with those blocks (Fig. 6b).

A perception of increased threat increased activation of the negativity subsystem^29,30^, which created a compensatory preference for risk-averse behaviour, e.g., one-foot checks, dwelling on Solid blocks.

The influence of threat on psychophysiological measures was assessed on an event-related basis. For these analyses, threat was highest when participants made a two-feet movement to a Crack block, which was visually indistinguishable from a Fall block. We found evidence of increased corrugator activity following a two-feet movement to Crack blocks and our analyses revealed that HN individuals exhibited greater corrugator reactivity following a two feet interaction to any block (Fig. 7a); this pattern may indicate greater negative affect for those individuals in response to uncertainty^34^ and anticipation of a virtual fall. It was also predicted that sympathetic activation would increase via elevated SCL during these high-threat interactions^44^, but this hypothesis was not supported by our data.

An interaction between trait neuroticism and period was apparent for zygomaticus activation during Risk Assessment (Fig. 7b). Interpretation of this effect is problematic because at least two lines of inference are possible. If positive affect is synonymous with activation of this muscle^35^, greater zygomaticus reactivity indicates positive anticipation for LN individuals, i.e., that the block would not crack. However, the reversal of this trend during the 751–1500 ms post-movement period (Fig. 7b) could indicate a positive response (e.g., relief) for HN individuals, when the block did not crack. Alternatively, increased zygomaticus activity could be interpreted as a grimace associated with negative affect^51^ for both LN and HN individuals. The absence of any differentiation between block type makes it impossible to interpret this effect with greater specificity.

With respect to previous research, our behavioural analyses replicated the association between negative emotional responses to threat and avoidant behaviour previously reported by Biedermann et al^20^. The absence of any significant increase of autonomic activation via SCL in the current study contrasts with earlier research^16,19,22^ where virtual height provoked greater SCL. However, the current study differed from existing work in a fundamental way and the lack of replication is unsurprising. The previous studies were designed to compare height with no height^16^ or different levels of height^19^ or being virtually elevated in an open vs. closed elevator^22^ whereas the experience of virtual height was held constant in the current investigation and our primary independent variables was the probability of a fall.

The results of the study can be summarized as follows: (i) room-scale VE allowed us to induce negative emotions that were seamlessly associated with naturalistic behavioural responses, (ii) by cross-referencing data, event-related psychophysiology could index momentary fluctuations following each interaction in the VE, and (iii) this methodology was utilized to achieve a graded activation of the negativity subsystem of the ESM. With respect to the latter, we demonstrated how threat induced compensatory behaviours to mitigate risk and increased negative affect during higher-risk interactions. Both patterns were enhanced for participants with higher trait neuroticism who had an implicit bias towards negative affectivity.

There were a number of areas where the methodology could be improved. A decision to maximise the autonomy of participants within the VE allowed individuals to ‘backtrack’ over previously activated blocks, which inflated the amount of interaction data derived from those participants; in hindsight, a mechanic should have been introduced to prevent backtracking. We also allowed participants to self-select speed of movement through the VE, which forced us to minimize time windows during the event-based analyses in order to eliminate overlap between successive post-movement and pre-movement periods. In the case of some variables, such as SCL, the 1500 ms post-event period may have blunted the sensitivity of the measure, i.e. SCL latency is 1–3s^52^.

The study demonstrated how room-scale VR can create a controlled environment capable of inducing embodied emotional experiences, which incorporate feedback from body posture as input to the emotional state^53,54^. At a more practical level, our results imply that behavioural data can be utilized to profile individual users of VR, in an implicit fashion similar to existing work utilising data from interactions on social media^55^, which has implications for data privacy as commercial VR systems are used at scale within the general population.

## Supplementary information

Supplementary Information 1.

Supplementary Information 2.
