# Supplementary material for: Assessment of threat and negativity bias in virtual reality
Source: Sci Rep. 2020 Oct 15;10:17338. doi: 10.1038/s41598-020-74421-1 (PMC7566621; doi:10.1038/s41598-020-74421-1)
Supplement: Supplementary file 2 — Supplementary Information 2. [file 41598_2020_74421_MOESM2_ESM.docx]

**SUPPLEMENTARY MATERIAL**

**Assessment of Threat and Negativity Bias in Virtual Reality**

Authors: Christopher Baker, Ralph Pawling, Stephen Fairclough

Attached – video file of virtual environment
